# The mediating role of self-efficacy in the association between social support and eHealth literacy among kidney transplant recipients

**DOI:** 10.3389/fpubh.2026.1864417

**Published:** 2026-07-08

**Authors:** Xu Liu, Xuange Sun, Xiaofei Li

**Affiliations:** 1 Transplantation and Hepatobiliary Department, The First Hospital of China Medical University, Shenyang, China; 2 School of Nursing, China Medical University, Shenyang, China

**Keywords:** EHealth literacy, kidney transplant recipients, mediation analysis, self-efficacy, social support

## Abstract

**Background:**

Kidney transplant recipients face complex self-management challenges that require a high level of health literacy, particularly in digital health contexts. However, the mechanisms underlying eHealth literacy in this population remain underexplored. This study aimed to examine the association between social support and eHealth literacy and to investigate the mediating role of self-efficacy in this relationship among kidney transplant recipients in China.

**Methods:**

A cross-sectional survey was conducted in June 2025 among 236 kidney transplant recipients at a tertiary hospital in northeastern China. Eligible participants had undergone kidney transplantation at the study hospital between January 2022 and May 2025. Standardized self-report questionnaires were used to measure social support, self-efficacy, and eHealth literacy. Mediation analysis was performed using the PROCESS macro for SPSS, adjusting for relevant covariates.

**Results:**

After adjustment for covariates, social support was significantly associated with self-efficacy (B = 0.351, *p* < 0.001) and eHealth literacy (total effect: B = 0.213, *p* < 0.001). When self-efficacy was included in the model, the direct association between social support and eHealth literacy remained significant (direct effect: B = 0.143, *p* < 0.001). The indirect effect through self-efficacy was also significant (B = 0.070, *p* < 0.001), indicating findings consistent with a partial mediation pattern.

**Conclusion:**

Among kidney transplant recipients, social support and self-efficacy were positively associated with eHealth literacy, and self-efficacy partly accounted for the association between social support and eHealth literacy. These findings suggest that perceived social support and self-efficacy may represent promising targets for interventions aimed at enhancing eHealth literacy and digital health engagement in this population.

## Introduction

Kidney transplantation remains one of the most effective therapeutic strategies for patients with end-stage renal disease (1). With advances in medical technology, transplant procedures and postoperative care have significantly improved, leading to prolonged graft survival and better patient outcomes. In recent years, clinical attention has increasingly shifted from surgical success to long-term postoperative management (2). Kidney transplant recipients typically experience enhanced autonomy, improved quality of life, and increased participation in daily and social activities, including returning to the workforce (3). However, sustaining graft function necessitates consistent engagement in complex self-care routines and long-term medical surveillance (4).

eHealth, defined as the use of internet-based and digital technologies to support healthcare delivery, has emerged as a valuable tool for promoting self-management, facilitating communication between patients and healthcare providers, and improving clinical outcomes (5, 6). In China, Internet healthcare services have expanded rapidly, and the number of Internet healthcare users has continued to increase in recent years (7). However, the growing availability of digital health services does not necessarily ensure that patients are able to effectively access, evaluate, and apply online health information. Recent evidence from China suggests that eHealth literacy remains suboptimal in some populations and is influenced by factors such as age, educational level, income, digital access, and frequency of eHealth use (8). These findings indicate that eHealth literacy has become an important public health issue in the context of digital healthcare development.

For kidney transplant recipients, eHealth literacy is particularly important because post-transplant care requires long-term medication management, graft function monitoring, infection prevention, and timely communication with healthcare professionals (9, 10). Through digital platforms, patients can access timely health information, virtual psychological support, and community interactions, which help mitigate the negative emotions caused by social isolation and enhance the patients’ self-management capabilities (8, 11). Previous studies have suggested that eHealth literacy is associated with digital health service use among transplant recipients, and eHealth-based interventions have shown potential for improving medication adherence in kidney transplant recipients (12). Nevertheless, evidence remains limited regarding the level of eHealth literacy among kidney transplant recipients in China and the psychosocial factors associated with it.

Social support has been widely recognized as a significant determinant of positive health behaviors and improved clinical outcomes (13). For kidney transplant recipients, social support is particularly important because long-term post-transplant management requires patients to understand complex care instructions, use digital health resources, manage medication regimens, and cope with uncertainty related to graft function and potential complications. Evidence from national studies indicates that individuals with greater social support exhibit enhanced abilities in accessing health-related information, managing disease-related symptoms, and maintaining better overall health and quality of life (14). Support from family members, friends, peers, and healthcare professionals may provide emotional reassurance, informational guidance, and practical assistance during long-term post-transplant management. Such support may also help patients engage more actively with digital health resources.

Self-efficacy denotes the belief in one’s ability to carry out tasks successfully and accomplish goals. It is a fundamental concept in health psychology and behavior change theories (15). Existing literature suggests that social support positively influences self-efficacy, which in turn facilitates the effective use of available support resources (16). In turn, higher self-efficacy may enable patients to more confidently seek, evaluate, and apply online health information, thereby contributing to better eHealth literacy. High self-efficacy has also been associated with greater engagement in health-promoting behaviors, such as medication adherence, regular follow-up, and effective use of health information (17). However, previous studies have mainly examined the independent associations of social support or self-efficacy with health-related outcomes, whereas few studies have explored whether self-efficacy explains the association between perceived social support and eHealth literacy among kidney transplant recipients.

**Figure 1:**
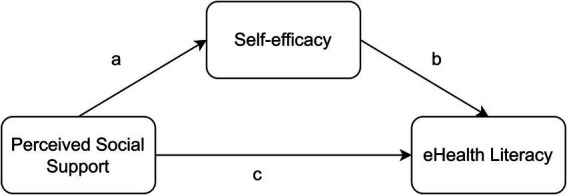
Conceptual model of the hypothesized mediation pathway.

According to Social Cognitive Theory, behavior change results from reciprocal determinism where personal, behavioral, and environmental factors influence one another bidirectionally (18). In the present study, perceived social support is conceptualized as an environmental resource, self-efficacy as a personal cognitive factor, and eHealth literacy as a digital health-related capability relevant to post-transplant self-management. Given the complexity of post-transplant care, perceived social support may help kidney transplant recipients develop greater confidence in managing their health and engaging with digital health resources; however, it remains unclear whether self-efficacy mediates the association between perceived social support and eHealth literacy among kidney transplant recipients. Therefore, this study aimed to examine the association between perceived social support and eHealth literacy among kidney transplant recipients and to determine whether self-efficacy mediates this association. The hypothesized conceptual model is presented in Figure 1. The findings may provide a theoretical basis for enhancing eHealth literacy and developing digital health-based self-management interventions for kidney transplant recipients, while also offering practical guidance for healthcare professionals in designing targeted digital health education strategies.

Drawing upon existing literature, the present study proposes the following hypotheses:

*H1*: Perceived social support is positively associated with eHealth literacy.

*H2*: Self-efficacy is positively associated with eHealth literacy.

*H3*: Self-efficacy mediates the association between perceived social support and eHealth literacy.

## Methods

### Participants

This cross-sectional study was conducted in June 2025 at a large tertiary hospital in northeastern China. Eligible participants were kidney transplant recipients who had undergone transplantation at this hospital between January 2022 and May 2025 and met the inclusion criteria without meeting any exclusion criteria. A total of 329 eligible recipients were invited to participate. Of these, 236 agreed to participate and completed the questionnaire, while 93 not to participate. No completed questionnaires were excluded, resulting in a final response rate of 71.7%.

Data were collected using both face-to-face and online questionnaires. The mode of questionnaire administration was determined by clinical circumstances. Participants who attended outpatient follow-up or were hospitalized during the survey period generally completed the questionnaire face to face, whereas those who were not present at the hospital and could be contacted remotely completed the online questionnaire. For face-to-face data collection, participants completed the questionnaires in a quiet and relatively private area of the transplant outpatient clinic or ward after routine follow-up care. Participants were seated comfortably and were given sufficient time to complete the questionnaire independently. To protect privacy, questionnaires were completed without the presence of unrelated individuals whenever possible, and participants were informed that they could pause or withdraw from the survey at any time. For online data collection, the questionnaire link was sent to eligible participants through a secure online survey platform named Wenjuanxing. The first page of the questionnaire provided information about the study purpose, voluntary participation, confidentiality, and contact details of the research team. If participants had questions or encountered ambiguous items while completing the online questionnaire, they could contact the researchers by telephone or online messaging. Trained researchers provided standardized clarifications regarding item wording or response options but did not guide or influence participants’ answers.

The survey included the General Information Questionnaire, eHealth·Literacy Scale, Perceived Social Support Scale, and Chronic Disease Self-Efficacy Scale. Eligible participants were selected according to the following inclusion criteria: (1) aged 18 years or older; (2) had undergone kidney transplantation at the study hospital between January 2022 and May 2025; (3) were clinically stable at the time of the survey; (4) were able to understand the study information and questionnaire items; and (5) voluntarily agreed to participate in the study. The ability to understand the study information and questionnaire items was confirmed during the informed consent and questionnaire administration process. Participants were excluded if they: (1) had active malignant tumors or other severe comorbid conditions that could substantially affect post-transplant self-management or questionnaire completion; (2) had undergone multi-organ transplantation; or (3) had obvious cognitive impairment, psychiatric disorders, or disturbance of consciousness that prevented independent questionnaire completion. These criteria were used to reduce clinical heterogeneity and ensure that participants could provide valid self-reported responses.

Ethical approval for this study was obtained from the Ethics Committee of the First Hospital of China Medical University (approval number: 2025[303]). The cross-sectional survey was conducted in June 2025 after ethical approval had been obtained. The period from January 2022 to May 2025 refers to the transplantation period used to identify eligible participants, not the data collection period. The study was conducted in strict accordance with the Declaration of Helsinki, ensuring confidentiality, minimizing harm, and obtaining informed consent from all participants.

## Measures

### Questionnaire for general information

The general information of this study was categorized into three aspects, including the basic information, the health-related information, and the Internet use. The basic information includes the age (<45, 45~, >60), gender (male, female), marriage (married, unmarried, divorced, widowed), body mass index (<18.5, 18.5~, >24.0), education level (junior high school and below, technical secondary school, high school, college degree, university and above), living status (living with others, living alone), employment status (employed, unemployed, retired), medical insurance (worker insurance, resident insurance, none), family per capita monthly income (<3,000, 3,000–6,000, 6,001–10,000, >10,000), and the financial burden (no, light, medium, heavy). The health-related information including the dialysis type (hemodialysis, peritoneal dialysis), dialysis duration (<5 years, ≥5 years), the 24 h urine volume (>2000 mL, ≤2000 mL), time since transplantation (<1 years, 1–<2 years, 2–<3 years, ≥3 years), self-rated health (poor, fair, good), total medication numbers (per day) (1, 2, 3, 4), and comorbidities of transplantation (0, 1, 2, 3, 4, 5). The Internet use includes the Internet use duration (<1 years, 1–5 years, 6–10 years, >10 years), frequency of Internet use for health-related purposes (hardly, occasionally, sometimes, always), frequency of searching health-related information (never, hardly, occasionally, sometimes, always), health information attitudes (do not trust, somewhat trust, trust, completely trust), and the number of online health information sources used. The latter variable was assessed using a multiple-response item asking participants to select the main online sources they used to obtain health information, including search engines such as Baidu, WeChat official accounts or mini-programs, short-video platforms such as Douyin, WeChat/QQ group sharing, medical forums, and other sources. The number of selected sources was summed for each participant and analyzed as a continuous count variable, with higher values indicating more diverse use of online health information sources.

### eHealth literacy

eHealth literacy was measured using the validated Chinese version of the eHealth Literacy Scale (eHEALS). The original eHEALS was developed as an 8-item instrument to assess individuals’ perceived knowledge, comfort, and skills in finding, evaluating, and applying electronic health information (5). Previous validation studies of the Chinese version have demonstrated good reliability and construct validity, and a three-factor structure has been reported in Chinese populations (19). The Chinese version used in this study includes eight items reflecting three domains: seeking and using online health information and services, interpreting online health knowledge, and appropriately applying online health information. Each item was rated on a 5-point Likert scale ranging from 1 (strongly disagree) to 5 (strongly agree), yielding a total score between 8 and 40. Higher scores indicate greater levels of eHealth literacy. Cronbach’s alpha for the scale in this study was 0.935, indicating excellent internal consistency.

### Self-efficacy

Self-efficacy was evaluated using the Chinese version of the Chronic Disease Self-Efficacy Scale (20, 21). Previous validation studies of the Chinese version have demonstrated good internal consistency and construct validity in Chinese populations (20). The scale assesses patients’ confidence in managing symptoms and common disease-management tasks. It employed a 1–10 rating scale with the total score ranges from 6 to 60, with higher scores indicating higher levels of self-efficacy. The CDSES showed a Cronbach’s alpha of 0.840 in this sample, indicating acceptable reliability.

### Social support

Perceived social support was measured using the Multidimensional Scale of Perceived Social Support (MSPSS), originally developed by Zimet et al. (22, 23). The MSPSS includes 12 items across three dimensions: family support, friend support, and support from significant others. The Chinese version of the MSPSS has been widely used in Chinese populations and has demonstrated good reliability and construct validity for assessing perceived support (24). Each item was rated on a 7-point Likert scale ranging from 1 (strongly disagree) to 7 (strongly agree), resulting in a total score ranging from 12 to 84. Higher scores indicate greater perceived social support. In this study, the MSPSS demonstrated high internal consistency, with a Cronbach’s alpha of 0.912.

### Sample size calculation

The sample size was initially estimated according to Kendall’s recommendation for multivariate analyses, which suggests at least 5 to 10 participants per variable. Considering the variables included in the planned analyses and a possible 10% rate of missing or invalid responses, the minimum required sample size was estimated to be approximately 149 participants.

Because the primary analysis involved a mediation model, methodological studies on mediation analysis were also considered, which indicate that sample size requirements depend on the expected effect sizes of the paths in the mediation model and the method used to test the indirect effect (25, 26). The final sample of 236 participants exceeded the minimum estimated requirement and was considered acceptable for the planned regression and mediation analyses.

Therefore, the final sample of 236 participants in this study exceeded the recommended threshold and was deemed sufficient for the planned statistical analyses.

### Statistical analysis

Data were analyzed using SPSS version 25.0. The normality of continuous variables was assessed through skewness and kurtosis tests (22). In this study, the skew value <2 and the kurtosis <7, indicating the data was normally distributed. Descriptive statistics were used to summarize participants’ general information. Univariate analyses were conducted to examine associations between demographic variables and eHealth literacy. Pearson correlation analysis was employed to explore the relationships among perceived social support, self-efficacy, and eHealth literacy.

A mediation model was tested using the PROCESS macro version 4.2, incorporating bootstrap resampling procedures. Covariates were selected based on previous literature, clinical relevance, and their associations with eHealth literacy in univariate analyses. The indirect effects were estimated using 5,000 bootstrap samples, and 95% confidence intervals (CI) were calculated. Effects were considered statistically significant if the confidence interval did not contain zero.

## Results

Descriptive statistics summarizing the recipients’ general information are shown in Table 1. Recipient age ranged from 20 to 71 years (Mean: 41.81, SD: 9.34), 138 (58.5%) were male, and most of the recipients (170, 72.0%) had undergone the hemodialysis before the transplantation. Meanwhile, most of the recipients (150, 63.6%) had undergone the dialysis less than 5 years. About half of the recipients (115, 48.7%) had used the Internet between 6 and 10 years. 47 (19.9%) of recipients rated their health as poor, 79 (33.5%) as fair, 110 (46.6%) as good.

**Table 1:** eHealth literacy among kidney transplant recipients.

Variables		*N* (%)	eHealth literacy	*t*/F	*p*
Age (years)	<45	155 (66.1)	24.95 ± 7.56	1.355	0.508
	45^~^	74 (31.4)	23.47 ± 7.51		
	>60	6 (2.5)	23.50 ± 7.15		
Gender	Male	138 (58.5)	24.33 ± 6.59	−0.390	0.697
	Female	98 (41.5)	24.66 ± 6.15		
Body mass index	<18.5	41 (17.4)	26.98 ± 6.12	3.932	0.021
	18.5^~^	105 (44.5)	23.84 ± 6.46		
	>24.0	90 (38.1)	24.07 ± 6.23		
Education level	Junior high school and below	60 (25.4)	22.52 ± 6.18	40.480	<0.001
	Technical secondary school or high school	51 (21.6)	23.12 ± 6.33		
	College degree	61 (25.8)	23.01 ± 6.48		
	University and above	64 (27.1)	28.77 ± 4.38		
Marriage	Married	155 (65.7)	24.22 ± 6.24	1.732	0.161
	Unmarried	58 (24.6)	24.52 ± 6.99		
	Divorced	17 (7.2)	27.53 ± 4.45		
	Widowed	6 (2.5)	21.83 ± 7.78		
Living status	With others	146 (61.9)	25.18 ± 6.56	2.204	0.029
	Living alone	90 (38.1)	23.31 ± 5.98		
Employment status	Employed	143 (60.6)	24.73 ± 6.60	0.379	0.685
	Unemployed	80 (33.9)	24.19 ± 6.14		
	Retired	13 (5.5)	23.38 ± 5.97		
Medical insurance	Worker insurance	121 (51.3)	24.73 ± 6.19	0.810	0.446
	Resident insurance	101 (42.8)	24.45 ± 6.75		
	None	14 (5.9)	22.43 ± 5.56		
Family per capita monthly income (yuan)	<3,000	56 (23.7)	24.88 ± 5.79	2.568	0.055
	3,000–6,000	77 (32.6)	25.57 ± 6.27		
	6,001–10,000	59 (25.0)	24.25 ± 6.96		
	>10,000	44 (18.6)	22.32 ± 6.23		
Financial burden	No	69 (29.2)	23.17 ± 6.75	2.035	0.110
	Light	36 (15.3)	25.81 ± 5.98		
	Medium	59 (25.0)	25.49 ± 6.44		
	Heavy	72 (30.5)	24.21 ± 6.07		
Dialysis type	Hemodialysis	170 (72.0)	24.52 ± 6.81	0.107	0.915
	Peritoneal dialysis	66 (28.0)	24.42 ± 5.28		
24 h urine volume (ml)	>2000	115 (48.7)	24.24 ± 6.27	−0.530	0.596
	≤2000	121 (51.3)	24.69 ± 6.54		
Dialysis duration (years)	<5	150 (63.6)	24.39 ± 6.56	0.265	0.791
	≥5	86 (36.4)	24.62 ± 6.13		
Time since transplantation (years)	<1	70 (29.7)	23.99 ± 7.03	1.101	0.347
	1–<2	109 (46.2)	24.20 ± 6.17		
	2–<3	50 (21.2)	25.26 ± 5.93		
	≥3	7 (3.0)	27.86 ± 6.26		
Self-rated health	poor	47 (19.9)	22.26 ± 6.77	4.635	0.011
	fair	79 (33.5)	24.25 ± 6.25		
	good	110 (46.6)	25.57 ± 6.13		
Total medications numbers (per day)	1	50 (21.2)	20.86 ± 6.23	12.022	<0.001
	2	64 (27.1)	23.27 ± 6.11		
	3	89 (37.7)	26.71 ± 6.02		
	4	33 (14.0)	26.24 ± 5.23		
Comorbidities of transplantation	0	24 (10.2)	27.17 ± 5.65	18.591	0.002
	1	44 (18.6)	23.14 ± 7.17		
	2	60 (25.4)	24.43 ± 7.14		
	3	79 (33.5)	23.38 ± 5.56		
	4	19 (8.1)	25.95 ± 5.37		
	5	10 (4.2)	29.90 ± 2.42		
Internet use duration (years)	<1	24 (10.2)	18.54 ± 5.70	19.397	<0.001
	1–5	34 (14.4)	21.88 ± 6.51		
	6–10	115 (48.7)	24.45 ± 5.62		
	>10	63 (26.7)	28.16 ± 5.59		
Frequency of internet use for health-related purposes	Hardly	21 (8.9)	18.71 ± 5.11	21.977	<0.001
	Occasionally	60 (25.4)	21.22 ± 5.99		
	Sometimes	72 (30.5)	25.44 ± 5.20		
	Always	83 (35.2)	27.43 ± 5.98		
Frequency of searching health-related information	Never	27 (11.4)	20.07 ± 6.70	55.680	<0.001
	Hardly	30 (12.7)	20.57 ± 6.45		
	Occasionally	72 (30.5)	23.24 ± 6.01		
	Sometimes	72 (30.5)	26.54 ± 4.77		
	Always	35 (14.8)	29.49 ± 4.45		
Health information attitudes	Do not trust	46 (19.5)	23.80 ± 6.40	2.334	0.075
	Somewhat trust	99 (41.9)	25.47 ± 5.73		
	Trust	57 (24.2)	24.56 ± 6.90		
	Completely trust	34 (14.4)	22.29 ± 6.99		
Number of sources used to obtain health information	1	76 (32.2)	22.78 ± 6.99	31.011	<0.001
	2	55 (23.3)	23.98 ± 5.61		
	3	80 (33.9)	24.56 ± 5.64		
	4	13 (5.5)	28.54 ± 5.46		
	5	12 (5.1)	32.42 ± 3.94		

Table 2 presented the results of the Pearson correlation analysis. The kidney transplantation recipients had an average eHealth literacy score of 24.47 ± 6.40. Their scores for perceived social support and self-efficacy were 55.72 ± 13.29 and 38.56 ± 11.74, respectively. Correlation analyses revealed significant positive relationships among eHealth literacy, social support, and self-efficacy (all *p* < 0.001). Specifically, eHealth literacy demonstrated a moderate positive correlation with social support (*r* = 0.495, *p* < 0.001) and self-efficacy (*r* = 0.533, *p* < 0.001). Additionally, social support was positively associated with self-efficacy (*r* = 0.419, *p* < 0.001).

**Table 2:** Correlation among of eHealth literacy, self-efficacy and social support.

Variables	Mean (SD)	1	2	3	4
1.eHealth literacy	24.47 (6.40)	1			
2. Self-efficacy	38.56 (11.74)	0.533**	1		
3. Social support	55.72 (13.29)	0.495**	0.419**	1	
4. Age	41.81 (9.34)	−0.246**	0.030	−0.056	1

^**^p < 0.001 (two-tailed). Correlations were Pearson correlation coefficients.

Mediation analysis (Model 4 of PROCESS Macro) was conducted to examine the role of self-efficacy in the relationship between social support and eHealth literacy. Using social support as the independent variable (X), eHealth literacy as the dependent variable (Y), and self-efficacy as the mediator (M), we controlled for relevant covariates and applied the Bootstrap method with 5,000 resamples to test the mediation effect. Age, gender, marital status, education level, family per capita monthly income, time since transplantation, total medication numbers per day, and comorbidities were included as covariates in the mediation model to control for potential confounding effects.

As shown in Table 3, based on the results of the mediation analysis using the regression-based mediation analysis, Model 1 demonstrated a significant positive association between social support and self-efficacy (B = 0.351, *p* < 0.001, 95% CI: 0.241, 0.461, *R*^2^ = 0.232). In Model 2, social support was significantly related to eHealth literacy (B = 0.213, *p* < 0.001, 95% CI: 0.164, 0.262, *R*^2^ = 0.482). In Model 3, both social support (B = 0.143, *p* < 0.001, 95% CI: 0.095, 0.190) and self-efficacy (B = 0.200, *p* < 0.001, 95% CI: 0.147, 0.253) were significantly associated with eHealth literacy (*R*^2^ = 0.585). These findings were consistent with a partial mediation pattern.

**Table 3:** Results of process distribution regression mediation effect test.

Outcome variable	Predictive variable	B	SEs	*β*	*t*	*p*	95% CI
Model 1							
Self-efficacy	Social support	0.351	0.056	0.394	6.312	<0.001	(0.241, 0.461)
Model 2							
eHealth literacy	Social support	0.213	0.025	0.438	8.539	<0.001	(0.164, 0.262)
Model 3							
eHealth literacy	Self-efficacy	0.200	0.027	0.366	7.462	<0.001	(0.147, 0.253)
	Social support	0.143	0.024	0.293	5.881	<0.001	(0.095, 0.190)

As shown in Table 4, the bootstrap analysis indicated a significant indirect association through self-efficacy. The indirect effect was 0.070, with a 95% confidence interval ranging from 0.044 to 0.099 that the direct and indirect effect was statistically significant. In addition, the confidence interval for the direct effect did not include zero, suggesting that the direct association between social support and eHealth literacy remained statistically significant.

**Table 4:** Results of the bootstrap mediation effect test.

Effect types	Paths	Effect	Bootstrap SE	Bootstrap 95% CI
Total effect	Social support → eHealth literacy	0.213	0.025	0.164, 0.262
Direct effect	Social support → eHealth literacy	0.143	0.024	0.095, 0.190
Indirect effect	Social support → self-efficacy → eHealth literacy	0.070	0.014	0.044, 0.099

## Discussion

To our knowledge, this study is among the first to examine the associations among perceived social support, self-efficacy, and eHealth literacy among kidney transplant recipients in China. The findings showed that perceived social support was positively associated with eHealth literacy, both directly and indirectly through self-efficacy. After controlling for covariates, this association remained stable. These findings are consistent with a partial mediation pattern, suggesting that self-efficacy may partly account for the association between perceived social support and eHealth literacy. Overall, the results highlight the importance of social and psychological resources in relation to eHealth literacy among kidney transplant recipients.

The findings were consistent with Hypothesis 1, showing that perceived social support was positively associated with eHealth literacy among kidney transplant recipients. This result is consistent with prior research showing that individuals embedded in stronger social networks are more likely to access health information, utilize digital resources, and engage in proactive health behaviors (27, 28). For kidney transplant recipients, social support may be particularly important because long-term post-transplant care requires continuous immunosuppressive medication management, graft function monitoring, prevention of complications, and regular communication with healthcare professionals (29, 30). Support from family members, peers, nurses, and transplant coordinators may help recipients understand medication instructions, evaluate the reliability of online health information, use telemedicine or online follow-up services, and manage anxiety related to graft function and long-term complications. Emotional support may reduce stress and uncertainty, thereby helping recipients engage more confidently with digital health platforms (12, 31). Informational and practical support may also reduce information overload, facilitate adaptive coping, and promote more active use of eHealth resources such as mobile health applications, online forums, and telemedicine services (32, 33). In addition, for recipients who are older, socioeconomically disadvantaged, or less familiar with digital technologies, assistance from supportive social networks may help reduce barriers to accessing and benefiting from digital health services (34, 35).

Second, the findings were consistent with Hypothesis 2 and 3, suggesting that self-efficacy may play a mediating role in the association between social support and eHealth literacy. These findings indicate a potential psychosocial pathway through which social support is associated with higher eHealth literacy: by fostering greater confidence in one’s ability to manage health-related tasks and make informed decisions, individuals are more likely to engage in and benefit from digital health resources (36). This result aligns with Bandura’s Social Cognitive Theory, underscoring self-efficacy as a key determinant of motivation, behavioral engagement, and goal attainment (18, 37). Within this theoretical framework, perceived social support can be viewed as an environmental resource, self-efficacy as a personal cognitive factor, and eHealth literacy as a digital health-related capability relevant to post-transplant self-management. For kidney transplant recipients, support from family members, peers, and healthcare professionals may strengthen confidence in managing health-related tasks, including the use of digital health resources (38, 39). This increased sense of self-efficacy, in turn, facilitates more active engagement with eHealth platforms, through which they can access essential health information, track medication adherence, and participate in remote monitoring activities that are vital for ensuring long-term transplant success (40). Therefore, healthcare interventions aimed at improving eHealth literacy should not only focus on providing access to technology but also prioritize the strengthening of recipients’ confidence in using digital health tools (41). This can be achieved through targeted social support initiatives, such as personalized training by healthcare professionals, emotional encouragement from family members and support from peer networks (42, 43). Such approaches can empower kidney transplant recipients to take a more proactive role in managing their health, thereby improving clinical outcomes and enhancing post-transplant quality of life (44).

The findings of this study have several implications. From a theoretical perspective, the results support the relevance of Social Cognitive Theory in understanding eHealth literacy among kidney transplant recipients, suggesting that perceived social support and self-efficacy are important psychosocial factors associated with digital health-related capability. From a clinical perspective, transplant nurses, transplant coordinators, and healthcare providers should not only provide transplant recipients with digital health information but also help them build confidence in seeking, evaluating, and applying such information. In routine follow-up care, healthcare professionals may assess recipients’ eHealth literacy, identify those with limited confidence in using digital health resources, and provide individualized guidance on reliable online information, medication management, telemedicine use, and symptom monitoring. Interventions that combine family or peer support, professional guidance, and self-efficacy enhancement may be useful for improving eHealth literacy and promoting digital health engagement in post-transplant self-management.

Several limitations of this study should be noted. First, the cross-sectional design limits the ability to infer causal relationships or temporal ordering among perceived social support, self-efficacy, and eHealth literacy. Although the mediation findings were statistically significant, they should be interpreted as associations rather than evidence of a causal pathway. Second, the sample was drawn from a single tertiary hospital in northeastern China, which may restrict the generalizability of the results to other kidney transplant populations, especially those in different cultural contexts or healthcare systems. Third, all variables were measured using self-report questionnaires, which may introduce recall bias and social desirability bias. Fourth, data were collected using both face-to-face and online questionnaires, and the mode of administration was determined by clinical circumstances rather than random assignment. Therefore, potential mode-related response bias cannot be fully excluded. Fifth, although data collection was conducted in June 2025, eligible participants had undergone kidney transplantation between January 2022 and May 2025. Differences in time since transplantation and changes in exposure to digital health services during this period may have influenced participants’ eHealth literacy. Finally, although several demographic and clinical covariates were adjusted for, unmeasured confounders, such as access to digital devices, quality of Internet access, prior experience with online health services, and family members’ digital support, may have influenced the observed associations.

Future research should further examine these associations using longitudinal or prospective designs to clarify the temporal relationships among perceived social support, self-efficacy, and eHealth literacy. Multicenter studies with larger and more diverse samples are also needed to improve the generalizability of the findings. In addition, intervention studies should explore whether strategies that enhance perceived social support and self-efficacy can effectively improve eHealth literacy and digital health engagement among kidney transplant recipients.

## Conclusion

This study found that perceived social support was positively associated with eHealth literacy among kidney transplant recipients, and self-efficacy partially mediated this association. These findings suggest that psychosocial resources, including perceived social support and self-efficacy, may be important factors related to patients’ ability to engage with health information in digital environments. Given the role of eHealth literacy in post-transplant self-management, strategies that strengthen social support and self-efficacy may represent promising approaches for enhancing digital health engagement among kidney transplant recipients. Future longitudinal and intervention studies are needed to further clarify the temporal relationships among these variables and evaluate effective strategies for improving eHealth literacy in this population.

## Data Availability

The raw data supporting the conclusions of this article will be made available by the authors, without undue reservation.
